# Radiolabeled PET/MRI Nanoparticles for Tumor Imaging

**DOI:** 10.3390/jcm9010089

**Published:** 2019-12-29

**Authors:** Ernesto Forte, Dario Fiorenza, Enza Torino, Angela Costagliola di Polidoro, Carlo Cavaliere, Paolo A. Netti, Marco Salvatore, Marco Aiello

**Affiliations:** 1 IRCCS SDN, Via Gianturco 113, 80143 Naples, Italy; eforte@sdn-napoli.it (E.F.); dfiorenza@sdn-napoli.it (D.F.); ccavaliere@sdn-napoli.it (C.C.); direzionescientifica@sdn-napoli.it (M.S.); maiello@sdn-napoli.it (M.A.); 2 Department of Chemical Engineering, Materials and Industrial Production, University of Naples Federico II, P.le Tecchio 80, 80125 Naples, Italy; an.costaglio@gmail.com (A.C.d.P.); nettipa@unina.it (P.A.N.); 3 Istituto Italiano di Tecnologia, IIT—Center for Advanced Biomaterials for Health Care, CABHC@CRIB, Largo Barsanti e Matteucci, 80125 Naples, Italy; 4 Interdisciplinary Research Center on Biomaterials, CRIB, University of Naples Federico II, P.le Tecchio 80, 80125 Naples, Italy

**Keywords:** multimodal imaging, hybrid imaging, positron emission tomography/magnetic resonance imaging (PET/MRI), nanotechnology, nanoparticles, in vivo imaging, 3D reconstruction

## Abstract

The development of integrated positron emission tomography (PET)/magnetic resonance imaging (MRI) scanners opened a new scenario for cancer diagnosis, treatment, and follow-up. Multimodal imaging combines functional and morphological information from different modalities, which, singularly, cannot provide a comprehensive pathophysiological overview. Molecular imaging exploits multimodal imaging in order to obtain information at a biological and cellular level; in this way, it is possible to track biological pathways and discover many typical tumoral features. In this context, nanoparticle-based contrast agents (CAs) can improve probe biocompatibility and biodistribution, prolonging blood half-life to achieve specific target accumulation and non-toxicity. In addition, CAs can be simultaneously delivered with drugs or, in general, therapeutic agents gathering a dual diagnostic and therapeutic effect in order to perform cancer diagnosis and treatment simultaneous. The way for personalized medicine is not so far. Herein, we report principles, characteristics, applications, and concerns of nanoparticle (NP)-based PET/MRI CAs.

## 1. Introduction

The growing technological development improved diagnostic imaging techniques allowing early disease detection and diagnosis [1,2,3,4]. Even if different imaging modalities are extensively used in clinical practice such as magnetic resonance imaging (MRI), computed tomography (CT), positron emission tomography (PET), single-photon emission tomography (SPECT), each one presents strong points and limits. Nuclear medicine imaging techniques (PET and SPECT) are highly sensitive (pM range) and quantitative but suffer from poor resolution (mm range) [5,6]; CT is widely available and can detect several pathologies through rapid examinations and easy three-dimensional (3D) reconstructions but radiation dose to the patient is a noticeable concern and it is limited in soft-tissue resolution [7]; MRI gives high resolution, anatomical information, and good soft-tissue contrast but has low sensitivity (mM) [8,9,10]. Table 1 summarizes imaging modalities and related features. Since no single imaging modality allows gathering all the necessary morphological and functional information, the combination of two or more imaging techniques, also called *multimodal imaging* or *hybrid imaging*, can offer synergistic advantages over any modality alone [11], overcoming its drawbacks and strengthening the peculiarities. The traditional approach was directed to the integration of a structural imaging modality (CT, MRI) with a functional highly sensitive imaging modality (PET/SPECT). Thus, firstly, PET/CT and SPECT/CT were introduced in clinical settings. The first PET/CT scanner was developed in 1998 by Townsend and colleagues [12] and was commercialized in 2001. It consists of a PET component independent from CT, and a single bed moves axially into the scanner while the patient sequentially performs CT and PET scans [13]. To date, PET/CT scanners completely replaced standalone PET scanners [14], exploiting anatomical reference and attenuation estimation from CT data. The success of PET/CT scanners inspired the feasibility of a PET/MRI scanner [15]. Three different configuration options were developed over the years [16]: the first consists of a sequential acquisition, similarly to PET/CT, where the patient undergoes firstly a MRI scan and later a PET scan; even if the MRI and PET components must be minimally modified, two consecutive acquisitions are performed without simultaneity. Temporal mismatches between PET metabolic data and MRI morphological information such as patient motion are the main weak points [7]. Nearly 15 years ago, some researchers working in preclinical settings analyzed the possibility of integrating a modified PET scanner into an MRI system. In Tubingen, Germany, an MRI-compatible PET scanner was inserted into a 3T clinical MRI scanner [8]; this system is suitable for preclinical studies or human brain imaging. The third option considers a first fully integrated whole-body PET/MRI system, with MRI-compatible photodiodes (avalanche photodiodes) and MR-based attenuation correction, and it became commercially available in 2010. It is worth noting that, in addition to anatomical information, MRI also provides functional information such as diffusion-weighted imaging (DWI), blood level oxygen-dependent (BOLD) imaging in functional MRI (fMRI), T_1_/T_2_ mapping, perfusion imaging and spectroscopy, and dynamic contrast-enhanced (DCE) imaging. PET and MRI can take reciprocal advantages: MRI anatomical data are useful for correction of the partial volume effect caused by PET [17], enable motion correction, improve arterial input function characterization for PET kinetic modeling, and are used as priors in PET iterative reconstruction; on the other hand, PET provides molecular information and highly sensitive quantification [18]. Furthermore, the simultaneous acquisition of fMRI data and metabolic PET information can investigate the coupling between metabolic demand and functional activity of the brain, since oxygen and glucose metabolism are strongly related to cerebral blood flow that delivers O_2_ and glucose to tissues [19]. High resolution and high tissue contrast, as well as multiparametric, functional, and quantitative imaging, supply complementary information for breast, head and neck [20], liver, musculoskeletal, and brain tumors [21] and heart [22] imaging. Hybrid imaging spread goes hand in hand with molecular imaging development, where molecular imaging stands for “in vivo” visualization, characterization, and measurement of biological processes at the molecular and cellular levels [23,24]. So far, various molecular imaging modalities were exploited not only for disease diagnosis, stratification, and treatment assessment [25] but also for image-guided therapy. Molecular imaging involves administration of imaging probes and detection of signals produced from the probes [26] and plays a key role in understanding important pathophysiological principles of diseases. In this context, personalized medicine aims to identify the adequate treatment and control its therapeutic efficacy. Suitable imaging probes are currently being developed and represent an exciting challenge for chemists and imaging scientists [27]. In this review, we focus on nanoparticle (NP)-based PET/MRI multimodal tracers in oncological imaging. A few of them were broadly tested in preclinical studies and show promising results in tumor detection, staging, and grading.

### 1.1. MRI Contrast Agents

In imaging, the term “contrast” refers to the capability of distinguishing between two adjacent structures; a contrast agent (CA) increases image contrast and highlights organs or blood vessels. The most common CAs used in X-ray or CT are iodinated and produce a direct effect on the image since they attenuate the X-ray beam, thereby increasing the signal intensity [20]; MRI CAs produce an indirect effect as they influence the relaxation times T_1_ and T_2_ of the neighboring water molecules. MRI CAs can act by reducing T_1_ or T_2_: the former are called T_1_-weighted CAs since they reduce the T_1_ relaxation time and brighten the resulting image, the latter are called T_2_-weighted CAs since they reduce T_2_ relaxation time and darken the resulting image [21]. T_1_-weighted CAs are paramagnetic lanthanide compounds like gadolinium (Gd^3+^) and manganese (Mn^2+^) chelates, while T_2_-weighted CAs are superparamagnetic agents like iron-oxide NPs [22]. CAs acting on both T_1_ and T_2_ relaxation times are called “dual mode” and are NP-based. MRI CAs available for the clinical practice are reported in Table 2.

The first requirement for a very efficient CA is a high relaxivity; this parameter indicates the efficiency in reducing T_1_ or T_2_ relaxation time of the surrounding water protons. Paramagnetic metal ions like Gd^3+^ cannot be used as CAs in their ionic form since their accumulation in specific tissues, for example, kidneys, liver, spleen, bone marrow, and the lymphatic system [28], causes toxicity. This challenge can be addressed by using chelators which hide the Gd ion through coordination bonds and are less likely to release it, conferring thermodynamic and kinetic stability and, therefore, less likely to induce toxicity. In particular, CAs based on Gd chelates are strongly associated with nephrogenic systemic fibrosis (NSF) in patients with renal impairment; the disease observed seems to be due to Gd release by chelating molecules in renal compartments [24]. In addition, recently, they were demonstrated to also accumulate in brain and kidneys in healthy patients [29,30].

To improve diagnostic efficacy and reduce the nephrotoxic effects, an ideal CA should be stable, biocompatible, not toxic, and specific; it should remain within the system for a sufficient time to produce desired effects, such as tumor accumulation for oncological imaging, but should also be excreted from the body to minimize unwanted effects of foreign materials within body. In addition, higher relaxivity suggests a lower CA dose in patients. Most CAs currently used (typically small Gd^3+^ ion chelates) lack in specificity because they are confined in the vascular space and do not accumulate in a specific tissue. It is not a coincidence that, in the last decade, CAs were refined by optimizing the relaxivity and developing amplification strategies aimed at increasing probe accumulation at the target site [25]. Moreover, the recent development of molecular and cellular imaging led to the recognition of NPs as MRI CAs.

### 1.2. Nanoparticles

In its first applications, hybrid PET/MRI was realized through the simultaneous administration of a mixture of MRI and PET probes, resulting in a cocktail of imaging agents causing high risk for the patient [38]. Additionally, this mixture could not guarantee an exact spatial and temporal correlation of the two imaging modalities due to the different biodistribution, pharmacodynamic and pharmacokinetic properties of the imaging agent. To overcome these limitations, nanoparticles (NPs) were proposed as delivery systems for different imaging agents to obtain bimodal probes for the simultaneous monitoring of both modalities. NPs are defined as particles with at least one dimension lying between 1 and 100 nm [39,40]. In recent years, very different NPs such as proteins, polymers, dendrimers, micelles, liposomes, viral capsids, metal oxides (iron-oxide NPs), zeolites, and mesoporous silicas were investigated, and very different shapes, such as spheres, cylinders (nanorods), and tubes were explored [24].

**Figure 1:**
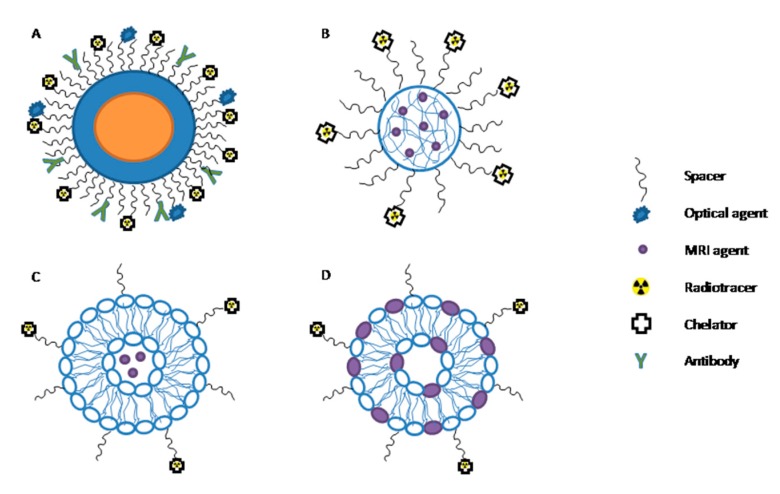
Multimodal nanoparticles. (**A**) Multimodal nanoparticle composed by a core (representing the magnetic resonance imaging (MRI) component) and a shell functionalized with an antibody. The positron emission tomography (PET) radiotracer is chelated and bound to the spacer. (**B**) A polymeric nanoparticle entrapping paramagnetic moieties is represented, where the PET radiotracer is chelated and bound to the spacer. (**C**) Liposomal formulation entraps paramagnetic moieties in the aqueous inner core, while the PET component is covalently linked to the spacer. (**D**) Liposomal formulation with paramagnetic ion inserted in the bilayer.

An NP-based PET/MRI bimodal probe constitutes three essential components: a carrier, a PET tracer usually represented by a positron emitter radioisotope characterized by high sensitivity (e.g., ^18^F-fluorodeoxyglucose), and an MRI component (e.g., gadopentetic acid (Gd-DTPA)), providing high tissue contrast and resolution. The MRI component can either work as a carrier itself (iron-oxide or gadolinium-oxide NPs) or can be a moiety bound to or entrapped in the carrier (e.g., Gd ions grafted onto NPs and polymeric matrices or biologically derived nanosized systems like apoferritin cages [41] and low-density lipoprotein (LDL) particles [42], respectively). Some possible configurations are reported in Figure 1.

As carriers, NPs offer a number of different design options, and the tailoring of their properties can be exploited to directly impact the in vivo fate of the resulting probe. Particle size, charge, core and surface properties, shape, and multivalency are the main features to be finely tuned in order to achieve a proper in vivo distribution, confer a targeting ability, and reduce toxicity of the NPs [43]. The hydrodynamic size determines the NP fate in the body, since vectors with a mean diameter smaller than 5 nm are usually eliminated by renal excretion, whereas larger particles (100 nm) are easily taken up by macrophages [44,45]. NP shape influences the internalization into cells that is relevant in cell tracking and labeling; for example, rod-like particles present higher internalization rates compared to spherical particles [46]. This phenomenon can be explained considering its similarity to rod-like bacterium internalization in nonphagocytic cells [47].

After NP injection into the bloodstream, they are rapidly coated by plasma proteins in a process called opsonization. The NPs are then recognized by plasma membrane receptors found on monocytes and macrophages and are, thus, taken up by the body’s main defense system, the reticuloendothelial system (RES), also known as the mononuclear phagocyte system (MPS). The liver, spleen, and bone marrow are rich in macrophages, thus becoming the most accessible organs to NPs [48]. For these reasons, NPs should be coated by adequate materials, to avoid nonspecific uptake by the RES [48,49,50] (stealth effect). In general, hydrophilic and neutral surfaces do not tend to interact with blood components (serum proteins); therefore, they are optimal for minimizing opsonization and clearance [51,52]. Since neutral polymers have no functional groups (amine, carboxyl, or hydroxyl) for ligand linkage, a further step of functional group activation is often mandatory. Another coating strategy employs hydrophilic bifunctional materials such as biphosphonate [53] or aluminum hydroxide [54]. NPs with biocompatible coating layers such as polymers (polyethylene glycol (PEG)), dendrimers, polysaccharides (dextran and chitosan), and polypeptides (serum albumin) can have enhanced properties including better stability in terms of agglomeration, biocompatibility, and solubility in water, along with low toxicity. The most used coating polymers are dextran, chitosan and, above all, PEG [41]. PEG is a hydrophilic, water-soluble, biocompatible polymer widely used to reduce opsonization and increase circulation time from seconds or minutes up to hours [55]. It is important to notice that surface modifications may have an impact on the superparamagnetic properties of iron-oxide NPs; for this reason, coating materials must be carefully chosen [56]. In particular, the nature and the thickness of the coating affect relaxivity. A more hydrophilic coating material results in more water molecules being retained for interacting with the magnetic centers; on the other hand, a thicker coating results in more protons being shielded from the magnetic field [57].

NP delivery to malignant cells can be achieved through both passive and active targeting. Passive targeting is due to the enhanced permeability and retention effect (EPR); since tumor vessels have larger fenestrations, the vascular permeability is higher, and NPs can easily extravasate in tumor tissue. Moreover, the inefficient lymphatic drainage contributes to NP retention in the tumor interstitial space [58]. Even though non-targeted NPs can accumulate in the tumor region due to the EPR effect, the lack of efficient lymphatic drainage generates an increase in interstitial pressure and, consequently, a drop in pressure gradient between the vessel and the extracellular space, causing nanoparticle stacking around the vessel wall [44,59]. For these reasons, there is a need for the development of NPs capable of efficiently and specifically targeting tumor cells [27]. The high NP surface-to-volume ratio helps to overcome this limitation since the NP surface can be functionalized through target-specific moieties that allow an active targeting of cancer cells.

Finally, multivalence refers to the ability to bind different imaging probes, targeting ligands, and therapeutic formulations. This feature is very important for multimodal and molecular imaging where a significant number of targeting probes are needed to track a specific biological path.

### 1.3. Radiolabeled Nanoparticles

Tracers currently used in clinical practice are labeled using positron emitters with a relatively low half-life time ranging from 2.037 to 109.8 min. Most of the radionuclides used for labeling are produced via a cyclotron. It generates a beam of accelerated protons and deuterons that are used to irradiate a target (e.g., ^14^N_2_ gas, ^20^Ne gas, ^18^O water or gas), thereby giving the desired radioisotope through a nuclear reaction. Table 3 shows the main radionuclides with related half-life time, the average energy of positron (β^+^), and means of production.

In PET clinical applications, ^18^F is one of the most suitable radionuclides for radiotracer synthesis since 97% of isotope decay is via positron emission [60], with a fairly low energy of positron emission (maximum 0.635 MeV) and an optimal half-life of 109.8 min, which is considered acceptable for chemical syntheses and favorable when investigating biological processes with a time frame longer than 100 min [61]. ^18^F-based radiotracers are essentially synthesized through two reactions: nucleophilic substitution or electrophilic substitution. Frequently, ^18^F is introduced to replace hydrogen in biomolecules. However, in terms of size, the van der Waals radius of ^18^F (1.47 Å) is closer to oxygen (1.52 Å) than that of hydrogen (1.20 Å) [62]; thus, ^18^F is generally obtained starting from water enriched with ^18^O through a nuclear reaction like ^18^O(p, n)^18^F.

PET radiotracers for cancer diagnosis can be grouped based on their target mechanism as follows:

- Radiotracers for the evaluation of glucose metabolism, such as fluorodeoxyglucose (FDG);
- Radiotracers for cell proliferation, such as ^18^F-fluorothymidine (FLT);
- Radiotracers for the evaluation of vascular perfusion, which include ^15^O-water and ^13^N-ammonia;
- Radiotracers for the evaluation of hypoxia, such as ^18^F-fluoromisonidazole (FMISO).

Other widely used radionuclides are ^68^Ga and ^64^Cu. In particular, ^64^Cu is gaining increasing interest for its theranostic potential [63]; during its decay, it emits both positron and Auger electrons allowing for both PET imaging and internal targeted radiation therapy. Indeed, Auger-emitting radionuclides that localize in the nucleus of tumor cells demonstrate a potential for cancer therapy. However, their biological effect is critically dependent on their sub-cellular (and sub-nuclear) localization [64] and on the DNA topology [65].

**Figure 2:**
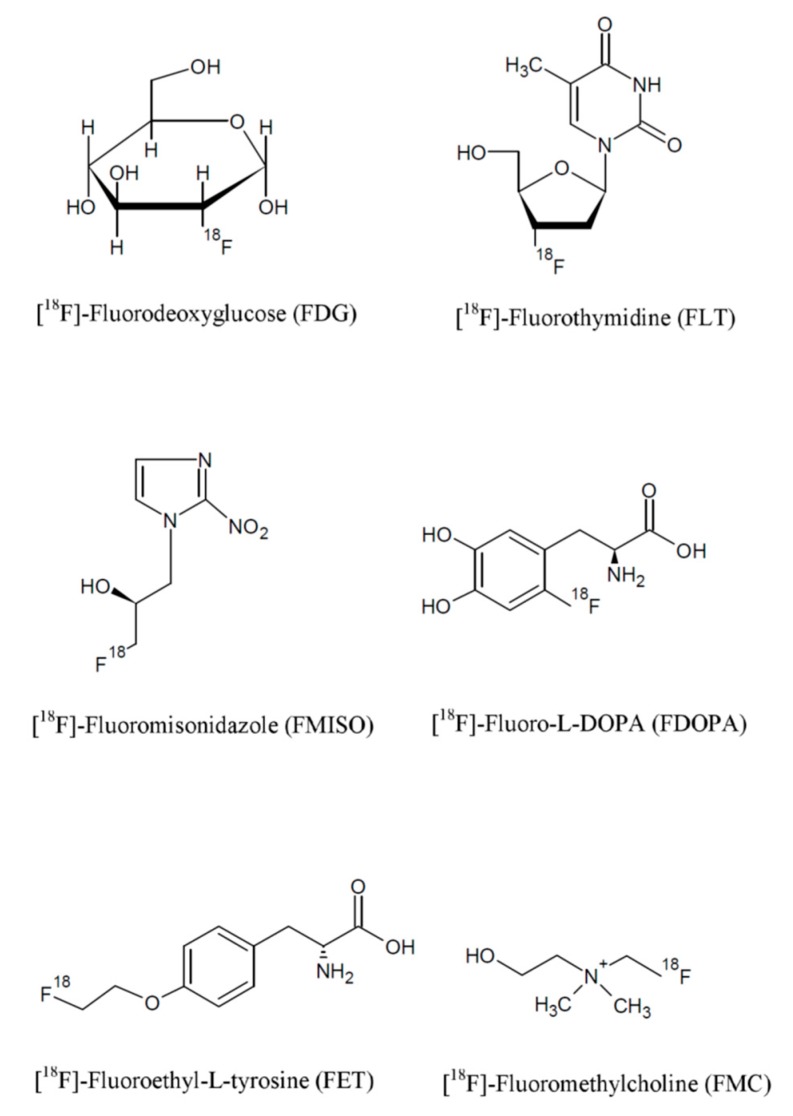
Chemical structure of fluorine-based radiopharmaceuticals.

The chemical structures of the most common radiotracers are reported in Figure 2.

NP radiolabeling with the abovementioned tracers can be achieved through different techniques. In the literature, the four following main strategies are reported [66]:

1. Complexation reactions of metallic radioisotope ions through coordination chemistry with the use of chelators;
2. Direct NP bombardment;
3. NP synthesis from radioactive and non-radioactive precursors;
4. Post-synthesis NP radiolabeling without the use of chelators.

The coordination chemistry approach is the most used since radioisotopes can be chelated by different molecules that are covalently bound directly to the NP surface. A strong linkage between the chelator coordinating the radioisotope and the NP surface is desired to assure the stability of the radiolabeling. It is worth noting that many exogenous chelators can currently only coordinate with certain radioisotopes, meaning that an effective chelator-based radiolabeling requires the selection of the best chelator for the isotope of interest [67]. In addition, the choice of the chelating agent should be such to minimize in vivo transchelation.

**Figure 3:**
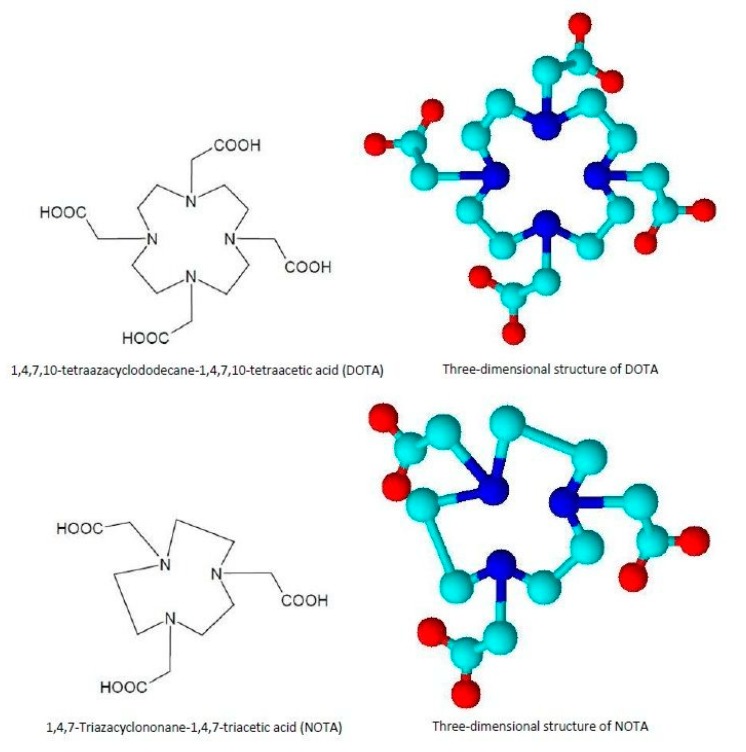
DOTA and NOTA chelators: chemical and three-dimensional structures. 1,4,7-triazacyclononane-N,N’,N’’-triacetic acid (NOTA) and 1,4,7,10-Tetraazacyclododecane-1,4,7,10-tetraacetic acid (DOTA).

New chelators for metallic radioisotopes were recently synthesized, including tetradentate acyclic chelators such as PTMS, esadentate acyclic chelators such as ethylenediaminetetraacetic acid EDTA or DTPA, and macrocyclic chelators such as 1,4,7-triazacyclononane-N,N’,N’’-triacetic acid (NOTA) and 1,4,7,10-Tetraazacyclododecane-1,4,7,10-tetraacetic acid DOTA [68], whose chemical structures are presented in Figure 3.

Recently, Laverman et al. reported the possibility of ^18^F chelation through an “Al–^18^F” complex, which carries out a coordination bond with the macrocyclic chelator NOTA [69].

Direct bombardment is achieved by direct irradiation of inorganic NPs with protons and neutrons to obtain radiolabeled NPs. Perez-Campana et al. demonstrated the nuclear reaction ^16^O(p, α)^13^N on Al_2_O_3_ NPs, where the radioisotope is incorporated in the inorganic NPs without any modification of the particle surface and morphology. Moreover, they demonstrated the stability of the radiolabeling by monitoring the in vivo signal after NP intravenous (i.v.) injection [70]. The main limitation of this approach is related to its application to functionalized NPs; the irradiation procedure may induce damages to the organic molecules conjugated onto the NP surface, causing the loss of their biological activity.

An alternative approach is the synthesis of radioactive NPs starting from radioactive and non-radioactive precursors. ^64^Cu is the most widely used radioisotope for this strategy thanks to which both organic and inorganic NPs can be obtained as liposomal ^64^Cu, [^64^Cu] CuS, or [^64^Cu] CuFe_3_O_4_ [71,72,73]. However, high temperatures and elevated incubation times are required for their production; thus, radiocontamination problems may arise.

Finally, post-synthesis NP radiolabeling seems to be a very promising chelator-free approach. However, both NP properties and chemical and physical interactions between NPs and the radioisotope have to be carefully taken into account. As an example, Chakravarty et al. produced a probe for dual MRI/PET imaging by ^69^Ge radiolabeling of superparamagnetic iron-oxide NPs (SPIONs). They were realized by exploiting the unique interaction between the NP surface and the radiotracer contact, overcoming all the limitations associated with the complex ^69^Ge coordination chemistry of traditional chelator-based methods [74].

Moreover, by exploiting the ability of some radiotracers to emit α and β particles, radiolabeled NPs can be used for radiation therapy in theranostic applications. These radiotracers, indeed, generate ionization in the atoms (mostly in water molecules), with the formation of free radicals and consequent damage to cellular DNA. As an example, liposomes containing α-emitters are widely described in the literature for their ability to improve the radionuclide circulation time and mediate its interaction with the biological environment [75,76,77]. Through this approach, it is possible to improve the ratio between radiation dose to tumor and normal tissues. Secondly, because of a better time to circulate, these formulations cause larger concentrations to diffuse within the tumor tissue and may, therefore, provide a less heterogeneous tumor dose [75].

### 1.4. PET/MRI Nanoparticles and Preclinical Applications

**Figure 4:**
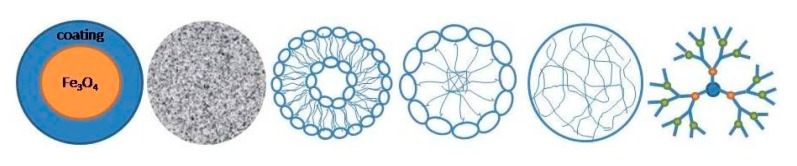
Typical nanocarriers: (from left) superparamagnetic iron-oxide nanoparticles, silica-based nanoparticles, liposomes, micelles, polymeric nanoparticles, and dendrimers.

NPs were extensively studied at a preclinical level as imaging probes for dual MRI/PET tumor imaging. According to the chemical composition of the core, NPs can be classified into inorganic and organic [78]. Inorganic NPs recently gained significant attention due to their unique physical and chemical properties. In particular, their chemical inertness, good stability, and the easiness of surface functionalization make inorganic NPs attractive for imaging of malignant tumors. However, their toxicity remains the main concern; it was demonstrated that iron-oxide NPs entering into cells through endocytosis show high toxicity because of their accumulation in endo-lysosomal compartments [59]. The most used carriers of this category are iron-oxide NPs and silica NPs. Common nanoconstructs are shown in Figure 4.

#### 1.4.1. Iron-Oxide Nanoparticles

Magnetic iron-oxide NPs, typically magnetite Fe_3_O_4_ and maghemite, γ Fe_2_O_3_, are broadly employed in MRI imaging especially for the liver, spleen, and bone marrow due, to their ability to shorten T_2_ and T_2_* relaxation times. According to their size, they can be categorized into *micrometer-sized paramagnetic iron oxide* (MPIO) (several micrometers), *superparamagnetic iron oxide* (SPIO) (hundreds of nanometers), *ultrasmall superparamagnetic iron oxide* (USPIO) (below 50 nm) [79], and *mono crystalline iron oxide* (MION) (representing a subset of USPIO ranging from 10 to 30 nm) [78,80].

The most common method for SPIO and USPIO synthesis is the reduction and coprecipitation reaction of ferrous and ferric salts in a basic aqueous media [81,82,83]. Resulting NPs are generally polydisperse and poorly crystalline; therefore, other preparation methods are often preferred, such as thermal decomposition and microwave synthesis [17]. Bare NPs are prone to agglomeration due to their high surface energy. In order to improve both colloidal and chemical stability, many polymeric coating materials were proposed, such as dextran, carboxymethylated dextran, carboxydextran, chitosan, starch, PEG, heparin, albumin, arabinogalactan, glycosaminoglycan, sulfonated styrene–divinylbenzene, organic siloxane, polyvinyl alcohol, poloxamers, and polyoxamines [84,85]. In addition, the polymeric corona is able to protect iron-oxide NPs, preventing erosion at acidic pH, lowering cytotoxicity [63]. The coating can be performed during the co-precipitation process, with the synthesis of the NPs occurring simultaneously to its coating [86,87] or post-synthesis, with the coating realized after the synthesis of the NPs [88,89]. Surface coating is a key factor for NP bioconjugation to biological ligands such as peptides or antibodies; therefore, it represents clinical potential for cancer imaging. Nevertheless, iron-oxide NPs have some important drawbacks. First of all, they act as negative contrast and, after administration, there is a loss of signal that makes medical evaluation less easy compared to T_1_ CA brightness. Moreover, the high susceptibility causes distortion artefacts and reduces the contrast-to-noise ratio [79]. Gd-based T_1_ agents are the most extensively and clinically used. Alloy materials were investigated to obtain more efficient T_2_ CAs because they are endowed with higher magnetic anisotropy [69], crystallinity, and relaxivity; thus, various bimetallic ferrite NPs named *magnetic engineered iron-oxide NPs*, such as CoFe_2_O_4_, MnFe_2_O_4_, and NiFe_2_O_4_, were tested [90].

There are several commercially available superparamagnetic iron-oxide NP formulations such as Feridex (Berlex,, Hanover, NJ, USA), Endorem (Guerbet, Villepinte, EU), and Resovist (Schering, EU, Japan). They are mostly used for liver and spleen tumors diagnosis [91], and the coating polymers are dextran for Feridex and Endorem, and an alkali-treated low-molecular-weight carboxydextran for Resovist [92]. Many preclinical studies were conducted to assess the iron-oxide NP potential as PET/MRI probes for cancer imaging exploiting both passive targeting (for lymph node mapping) and active targeting strategies (mainly through RGD (Arg–Gly–Asp) conjugation).

Thorek and coworkers [93] prepared ^89^Zr radiolabeled iron-oxide NPs (ferumoxytol) to visualize the axillary and brachial lymph node drainage in healthy wild-type mice. In detail, the iron-oxide core was surrounded by a semisynthetic polysaccharide coating of polyglucose sorbitol carboxymethylether, and desferrioxamine was used as a chelator. In the same study [93], after intraprostatic administration in Hi-Myc transgenic mice bearing invasive prostatic adenocarcinoma, PET/MRI imaging delineated draining nodes in the abdomen and the inguinal region, in addition to prostatic ones.

In 2019, Madru et al. [94] proposed a new, time-efficient, chelator-free conjugation of ^64^Cu on PEGylated SPIONs for PET/MRI detection and localization of sentinel lymph nodes (SLNs) in C57BL/6J mice. The stability of radiolabeling up to 24 h and NP accumulation in the SLN were demonstrated through a biodistribution study. Lymph nodes metastases are important markers for cancer staging and treatment, and their localization can be useful in presurgical planning.

Xie and colleagues [95] encapsulated iron-oxide NPs, after modification with dopamine, into human serum albumin (HSA) matrices and labeled them with Cy5.5 dye and ^64^Cu-DOTA. NPs were injected into a U87MG xenograft mouse model; PET and NIRF imaging showed a higher signal-to-noise ratio compared to MRI because of their higher sensitivity. On the other hand, MRI scans post NP injection showed a clear inhomogeneous distribution thanks to their high spatial resolution. These findings were confirmed by histological studies. The HSA shell conferred prolonged circulation time and lower macrophage uptake rate. Such NPs are suitable for theranostic applications if co-loaded with drug molecules.

An active targeting probe was developed by Lee and coworkers [96] who conjugated RGD to ^64^Cu radiolabeled iron-oxide NPs. As a coating material, polyaspartic acid was chosen since it exposes both carboxyl groups interacting with NPs and amine groups useful for DOTA and RGD conjugation. Imaging was performed on a U87MG mouse model, and both PET and MRI confirmed that the accumulation of NPs was mediated by α_v_β_3_ integrin binding. Kim and colleagues [97] injected ^68^Ga labeled iron-oxide NPs into BALB/c nude mice bearing colon cancer (HT-29) cells, using oleanolic acid as a tumor-targeting molecule. This ligand was shown to inhibit colon cancer cell proliferation, as well as induce apoptosis and cancer cell death. Binding assays and histological studies confirmed the tumor uptake of NPs thanks to oleanolic acid affinity for HT-29 cancer cells. PET/MRI scans provided high-quality images and precise quantification of the tumor area.

#### 1.4.2. Silica-Based Nanoparticles

Silica-based NPs are widely applied in drug delivery, bio-imaging, and cell targeting as they are considered an ideal biocompatible matrix to integrate imaging probes. There are two major classes of silica-based NPs: solid (SiNPs) and mesoporous (MSNs). SiNPs are extensively used as optical imaging agents, while MSNs are often used in CT, MRI, PET, molecular, and multimodal imaging. MSNs are synthesized by a surfactant templated sol–gel method [98] and have attractive properties such as an extremely large surface area, a tunable structure in terms of size, morphology, and porosity, and ease of functionalization through synthetic approaches [99]. In bimodal PET/MRI imaging, MSNs are used as a coating material for metallic NPs or as carrier for MRI CAs and PET radioisotopes. CAs can be encapsulated in channels and protected from the environment, together with drugs or genes for theranostic purpose. A porous silica shell improves MRI contrast enhancement since the pores allow intimate contact between water molecules and the iron-oxide NPs [100]. We report two examples of MSNs in PET/MRI for cancer imaging. Burke and colleagues [101] described silica-coated iron-oxide-based nanorods radiolabeled with ^68^Ga. In detail, nanorods were coated with various ratios of a siloxane-terminated tetraazamacrocycle (siloxane-DO3A) and a siloxane PEG derivative. Nanorods offer some advantages over nanospheres such as improved T_2_ MRI contrast and direct uptake in the liver via phagocytosis. Moreover, thanks to the silica coating, a macrocyclic chelator for highly stable radiolabeled nanoconstructs was not required. Huang and coworkers [102] reported a mesoporous silica-based triple modal imaging nanoprobe to map and track tumor metastatic sentinel lymph nodes (T-SLNs). In this system, three imaging probes including near-infrared (NIR) dye ZW800, T_1_ CA Gd-DTTA, and the positron-emitting radionuclide ^64^Cu were integrated into MSNs via different conjugation strategies. PET and MRI imaging probes were located on the surface and in the mesoporous channel of NPs. A faster uptake rate and higher uptake of the multifunctional MSN probes were observed in T-SLNs compared with normal SLNs, confirming the feasibility of these MSN probes as CAs to map SLNs and identify tumor metastasis. Images revealed that NP accumulation in T-SLNs was much higher than in normal controlateral SLNs (N-SLNs), where almost no signal was observed.

#### 1.4.3. Organic Nanoparticles

Over the last decade, a number of organic NPs, such as dendrimers, polymeric micelles, liposomes, and proteins were used in various applications for cancer diagnosis. These organic NPs carry imaging moieties such as radionuclides and show potential for tumor diagnosis [78]. Liposomes are spherical phospholipid bilayers similar to a cell membrane. Phospholipids are amphiphilic molecules as they have a hydrophilic head group and two hydrophobic tails; thus, they present an inner aqueous compartment that can encapsulate hydrophilic molecules, while hydrophobic agents can be inserted in the lipid shell. Liposomes can be classified by size or by the number of bilayers. In fact, they can also present more than one bilayer; these multilamellar constructs are characterized by an onion structure where each bilayer of phospholipids is separated from the adjacent by a water layer [82]. Among the various protocols for preparing liposomes with different size and number of layers, the most established are based upon sonication and extrusion [82]. Functional moieties can be attached on the bilayer membrane surface. They are biocompatible, non-toxic, and biodegradable, and they are extensively used for drug delivery. After PEGylation, the blood circulation time of liposomes can be prolonged for sustained release or targeted delivery of imaging and therapeutic agents [44]. Liposomes can exploit the EPR effect or active targeting with antibodies, peptides, and vitamins to reach cancer cells [103]. To date, several liposomal formulations were approved for cancer therapy, mainly loaded with doxorubicin, and treatment of infections such as fungal infections; a few anticancer-loaded liposomes are currently undergoing clinical trials [104].

In MRI imaging, liposomes can be used as a coating material to prevent iron-oxide NPs from aggregating and to target tumor cells. They are an excellent platform for multimodal imaging and theranostic application. As an example, Malinge et al. [85] realized magnetic liposomes by incorporation of iron-oxide NPs in the liposomal aqueous core. Liposomes were radiolabeled through a ^68^Ga-based radiotracer allowing a dual-modality tracking of particle in vivo distribution through MRI and PET imaging; in addition, glucose was grafted onto the NP surface. On U87MG-bearing mice, the magnetic characteristic of the liposomes and the superficial presence of the glucose enabled a dual tumor-targeting mechanism. Through an external magnet, particles were driven in the tumoral region, and the Warburg mechanism allowed their preferential interaction with tumoral cells. In addition, this study confirmed the role of the lipid bilayer in regulating the exchange of water molecules from the external environment to the aqueous core and the consequent increase of the iron-oxide NPs relaxivity r_2_, improving MRI performance. In another study, Li and colleagues [105] constructed a multifunctional theranostic liposomal drug delivery system; liposomes encapsulated doxorubicin and were conjugated with Gd-DOTA for MRI and IRDye for near-infrared fluorescence. Liposomes were also radiolabeled with ^99m^Tc and ^64^Cu for SPECT and PET imaging. After intratumoral injection, MR images displayed, with high resolution, the micro-intratumoral distribution of the liposomes in squamous cell carcinoma of head and neck tumor xenografts in nude rats. NIR fluorescent, SPECT, and PET images confirmed MRI findings. In addition, these multifunctional liposomes have the potential for the accurate monitoring and in vivo delivery of liposomal chemotherapeutic drugs or therapeutic radionuclides such as ^186^Re/^188^Re. Mitchell et al. [106] prepared liposomal formulations with short *n*-ethylene glycol spacers of varying length; multifunctional imaging was gained through a chelator (DOTA) in the head group of lipids, thereby chelating Gd^3+^ for MRI, ^111^In for SPECT, and ^64^Cu for PET. Compared to conventional PEG shielded liposomes (DSPE-PEG2000), this system showed good cellular internalization in tumor cells and similar distribution and blood half-lives. Abou and colleagues [107] radiolabeled preformed paramagnetic (Gd) liposomes with ^89^Zr (positron emitter). The authors used a chelator-free strategy thanks to the radiometal affinity for the lipid phosphate head groups; this dual mode CA was conjugated with octreotide to selectively target neuroendocrine tumors via human somatostatin receptor subtype 2 (SSTr2). MR and PET images revealed significantly greater accumulation of octreotide liposomes to SSTr2-expressing cells compared to control liposomes.

Like liposomes, micelles are also characterized by a core/shell structure but, unlike liposomes, the core can also be hydrophobic while the shell is hydrophilic. Micelles can be made of nonionic surfactants (surfactant micelles) or of amphiphilic block copolymers (polymeric micelles). In polymeric micelles, the length of the hydrophilic block exceeds that of the hydrophobic one, thus resulting in spherical shapes [88]. Polymeric micelles were greatly investigated for delivering hydrophobic drugs; moreover, they have a smaller size compared to liposomes, and the hydrophilic shell reduces interactions with macrophages [108]. Because of the hydrophilic nature of CAs, they can be bound to the hydrophilic blocks or covalently conjugated to the hydrophobic lipid chain in order to be incorporated into micelles [109]. A special group of polymeric micelles can be synthesized by the conjugation of water-soluble copolymers with lipids constituting the hydrophobic blocks (such as polyethylene glycol–phosphatidyl ethanolamine, PEG–PE). The main feature that makes PEG–lipid micelles attractive for diagnostic imaging applications is their size [88]; in fact, due to the lipid bilayer curvature limitation, it is not possible to prepare liposomes that are smaller than a certain minimal diameter (usually, 70–100 nm) [110]. Such a vector was realized by Trubetskoy and colleagues [111]; Gd-DTPA-PE and ^111^In-DTPA-SA were incorporated into 20-nm PEG–PE micelles to visualize lymph nodes during percutaneous lymphography using gamma scintigraphy and MRI imaging in rabbits. A recent study by Starmans et al. [112] provided a PET/MRI dual imaging polymeric micellar system consisting of self-assembling amphiphilic diblock copolymers functionalized with ^89^Zr deferoxamine and Fe^3+^ deferoxamine. In vivo PET and MRI images clarified tumor visualization thanks to the EPR effect. However, both liposomes and micelles are unstable, especially in the presence of serum, and, for this reason, many authors crosslink them to achieve better stability [94,95].

Dendrimers are a group of highly branched spherical polymers with a tree-like internal structure. They are characterized by an inner core surrounded by a number of branches called *generations*. Depending on the number of generations, they vary in size and molecular weight. CAs or drugs can be encapsulated in the inner spaces or anchored on the external terminations [113]. To date, dendrimers as dual modal agents are used for MRI and fluorescence [114,115], optical imaging and nuclear medicine [116], CT, and MRI [117].

However, few studies on positron-emitting radionuclide-labeled dendrimers were reported [118,119], and when such dendrimer platforms are used to develop PET/MRI or SPECT/MRI agents, it is challenging to achieve precise control of radioisotope loading into specific chelating moieties [120]. Indeed, to our knowledge, studies about dendritic formulations for combined PET/MRI remain to be published.

In recent years, the biomimetic approach gained increasing interest in the scientific community, and many scientists are trying to mimic what naturally occurs in the body in order to obtain more biocompatible and biodegradable materials for medical applications. The crucial idea behind the biomimetic approach is that a biopolymer naturally occurring in living organs can be modified for diagnostic and therapeutic purposes, improving probe efficiency and reducing immunogenicity and inflammatory potential. Biological polymers such as alginate, hyaluronic acid, and chitosan, as well as proteins, antibodies, enzymes, lipoproteins, and viral capsids (protein cages), are becoming very attractive for diagnostic and therapeutic applications [121].

Maham et al. synthesized engineered platforms for drug delivery systems of different types and shapes (NPs, microspheres, films, minirods, hydrogels) using gelatin, albumin, collagen, elastin, ferritin/apoferritin, gliadin, casein, zein (corn protein), whey protein, and soy protein [103] highlighting promises and challenges.

Vecchione et al. [122] proposed a fully biocompatible platform for dual MRI/PET imaging with improved relaxometric properties. The core–shell nanocarriers made of chitosan and hyaluronic acid entrapped Gd-DTPA, boosting its relaxometric properties up to five times, and carried the adsorbed ^18^F-FDG without any modification of both FDA-approved CAs.

Fan and coworkers [123] produced a water-soluble melanin NP formulation; this system, after PEGylation, naturally bound ^64^Cu and Fe^3+^ for PET and MRI imaging, and its surface was functionalized with RGD. Shukla et al. [124] proposed a virus-based synthesis, where bacteriophages and plant viruses were used as a scaffold to carry ^18^F and iron oxide or Gd^3+^. These virus-based NPs resulted homogenous and monodisperse, representing a promising delivery system for CAs. However, there are still several open concerns related to their immunogenicity and loading efficiency. In Table 4, a comprehensive overview of nanoparticulate constructs used in PET/MRI imaging and related properties is provided.

## 2. Conclusions and Perspectives

Molecular imaging and multimodal imaging are current topics extensively investigated by researchers and scientists. Since each diagnostic modality presents advantages and drawbacks, no single technique is able to provide a comprehensive overview of morphological, functional, and metabolic processes underlying tumors. Thus, a deep analysis was conducted on hybrid imaging, and several multimodal scanners are now routinely used in clinical practice among which PET/CT and PET/MRI are the most popular. The complementary information simultaneously obtained by PET and MRI offers new insights into disease diagnosis and treatment. As an example, dynamic contrast-enhanced MRI and PET with perfusion tracers are used to assess the tumor perfusion. A dual CA in a single probe allows a really simultaneous acquisition, and the co-localization of the two CAs guarantees a temporal and spatial correlation of the two imaging modalities. NPs can be used in PET/MRI as CAs for cancer imaging and, in order to gather anatomical and pathological information, their features must be properly adjusted: size, shape, charge, coating, and multivalency. Nevertheless, active targeting opened new pathways through the possibility of NP accumulation at the pathological site and, therefore, quantification is possible also on low-sensitivity techniques such as MRI. The impact of this approach can also be huge in the theranostic field since cancer imaging, diagnosis, and characterization can be used to gather important information about drug release, efficient therapy, and monitoring of response to treatment. Ideal candidates for a specific treatment could be so individuated, and personalized medicine can offer better results and faster healing. Even though, in the last few years, a variety of multimodal probes were produced, only few of them are approved for clinical use. Many challenges must be solved to promote NP clinical translation for both diagnostic and theranostic purposes. A multidisciplinary approach is necessary in order to focus on diagnostic applications and understand biomolecular processes at the basis of several pathologies. We expect that prevention, early diagnosis, patient management, and treatment could be improved such that a single performance can provide a comprehensive examination from which all essential parameters can be derived. In this perspective, multimodality plays a key role, and NPs can display their potential.

## Figures and Tables

**Table 1:** Molecular imaging modalities.

Imaging Technique	Source of Imaging	Spatial Resolution	Tissue Penetration Depth	Sensitivity	Agent	Ref.
Magnetic resonance imaging (MRI)	Radio wave	25–100 µm	No limit	mM to µM (low)	Para-(Gd^3+^) or superparamagnetic (Fe_3_O_4_) materials	[31]
Single-photon emission computed tomography (SPECT)	γ-ray	6–7 mm	No limit	pM (high)	Radionuclides (^99m^Tc,^201^Tl,^111^In,^131^I, ^123^I, ^67^Ga)	[32]
Positron emission tomography (PET)	γ-ray	1–2 mm	No limit	pM (high)	Radionuclides (^18^F,^11^C,^13^N,^15^O,^124^I,^64^Cu, ^68^Ga)	[33]
Computed tomography (CT)	X-ray	50–200 µm	No limit	n.c.	High-atomic-number atoms (iodine, barium sulfate)	[34]
Ultrasonography (US)	Ultrasounds	50–500 µm	mm to cm	n.c.	Microbubbles	[35]
Optical fluorescence imaging	Visible or near-infrared light	In vivo 2–3 mm in vitro µm	<1 cm	nM to pM (medium)	Fluorescent dyes, quantum dots	[36,37]

n.c., not well characterized.

**Table 2:** Magnetic resonance imaging contrast agents.

Brand Name	Active Substance	Chemical Name	Molecular Structure	Company	Current Status
Omniscan	Gadodiamide	Gd-DTPA-BMA	Linear, non-ionic	GE Healthcare	Suspended
OptiMARK	Gadoversetamide	Gd-DTPA-BMEA	Linear, non-ionic	Mallinckrodt	Suspended
Magnevist	Gadopentetic acid	Gd-DTPA	Linear, ionic	Bayer	Suspended
MultiHance	Gadobenic acid	Gd-BOPTA	Linear, ionic	Bracco	Only for liver scans
Primovist	Gadoxetic acid	Gd-EOB-DTPA	Linear, ionic	Bayer	In use
ProHance	Gadoteridol	Gd-HP-DO3A	Cyclic, non-ionic	Bracco	In use
Gadovist	Gadobutrol	Gd-BT-Do3A	Cyclic, non-ionic	Bayer	In use
Dotarem	Gadoteric acid	Gd-DOTA	Cyclic, ionic	Guerbet	In use

**Table 3:** Principal radionuclides and related features.

Radionuclide	Half-Life Time *	Electronic Emission Energy β+	Production
^11^C	20.385 min	386 keV	Cyclotron
^13^N	9.965 min	492 keV	Cyclotron
^15^O	122.24 s	735 keV	Cyclotron
^18^F	109.77 min	250 keV	Cyclotron
^64^Cu	12.701 h	655 keV	Cyclotron or reactor
^68^Ga	67.629 min	836 and 353 keV **	Generator

* The values were obtained from the database of the National Nuclear Data Center (NNDC) at Brookhaven National Laboratory, Upton NY, USA. ** Mean energy of the β spectrum.

**Table 4:** Overview of multimodal PET/MRI nanoparticles.

Nanostructure	MRI Component	PET Component	Chelator	Other	Biological Target	Ref.
Bacteriophages/plant viruses	Iron oxide/Gd^3+^	^18^F			Passive targeting	[124]
Hyaluronic acid + chitosan	Gd-DTPA	^18^F-FDG	No chelator		Passive targeting	[122]
Iron oxide + ligands (–NH_2_ –COOH)	Iron oxide	^11^C	No chelator		Passive targeting	[125]
Iron oxide + micelle + PEG	Iron oxide	^64^Cu	DOTA		Passive targeting	[126]
Iron oxide + dextran	Iron oxide	^64^Cu	DTCBP		Passive targeting	[53]
Iron oxide + HSA	Iron oxide	^64^Cu	DOTA	Cy5.5	Passive targeting	[95]
Iron oxide + mannose	Iron oxide	^68^Ga	NOTA		Passive targeting	[127]
Iron oxide + micelle + PEG	Iron oxide	^68^Ga	NOTA		Oleanolic acid	[97]
Iron oxide + PASP	Iron oxide	^64^Cu	DOTA		RGD *	[96]
Iron oxide + PEG	Iron oxide	^64^Cu	NOTA	Au	Anti EGFR affibody	[128]
Iron oxide + PLGA + lipids + PEG	Iron oxide	^64^Cu	DOTA		Passive targeting	[129]
Iron oxide + polyglucose	Iron oxide	^89^Zr	Desferrioxamine		Passive targeting	[93]
Iron oxide + PEG	Iron oxide	^64^Cu	No chelator		Passive targeting	[94]
Iron oxide + silica + PEG	Iron oxide	^68^Ga	DO3A		Passive targeting	[101]
Liposome	Iron oxide	^68^Ga	NODA	Glucose	External magnetic field + Warburg effect	[130]
Liposome	Gd-DTPA	^89^Zr	no chelator		Octreotide	[107]
Liposome	Gd ^3+^	^64^Cu	DOTA	IRDye–doxorubici n–^99m^Tc	Passive targeting	[105]
Liposome + nEG spacer	Gd ^3+^	^64^Cu	DOTA	^111^I–fluorescein	Passive targeting	[106]
Melanine NP + PEG	Fe^3+^	^64^Cu	No chelator		RGD *	[123]
Mesoporous silica NP	Gd^3+^	^64^Cu	DOTA	ZW800	Passive targeting	[102]
Micelle	Fe^3+^	^89^Zr	Desferrioxamine		Passive targeting	[112]
MnMEIO/iron oxide + Al(OH)_3_	MnMEIO/iron oxide	^18^F/^64^Cu	No chelator/DTCBP		Passive targeting	[54]
MnMEIO + SA	MnMEIO	^124^I	No chelator		Passive targeting	[131]
Silica NP	Gd^3+^	^68^Ga	DOTAGA/NODAGA		Passive targeting	[132]

* HSA = human serum albumin; RGD = Arg–Gly–Asp; PEG = polyethylene glycol; PLGA = polylactic-*co*-glycolic acid; nEG = *n*-ethylene glycol spacers; MnMEIO = Mn-doped magnetism engineered iron oxide; PASP = polyaspartic acid; DTCBP = dithiocarbamatebisphosphonate; EGFR = epidermal growth factor receptor; NOTA = 1,4,7-triazacyclonane-1,4,7-triacetic acid; DOTA = 1,4,7,10-tetraazacyclododecane-1,4,7,10-tetraacetic acid; DOTAGA = 1,4,7,10-tetraazacyclododecane-1-glutaric anhydride-4,7,10-triacetic acid; NODAGA = 2,2′-(7-(1-carboxy-4-((2,5-dioxopyrrolidin-1-yl)oxy)-4-oxobutyl)-1,4,7-triazonane-1,4-diyl) diacetic acid; DO3A = 1,4,7-tris(carboxymethylaza)cyclododecane-10-azaacetylamide. FDG = fluorodeoxyglucose.
